# Chloroform

**Published:** 1850-10

**Authors:** 


CHLOROFORM.

In the Dublin Quarterly Journal, August, 1850, Dr. Beatty publishes an excellent article on the use of Chloroform, which we intended to quote among our extracts in the present number of the *Journal*. But, as we had no space to spare for selections, we must be content with the following remarks of Dr. Beatty, on a paper, read before the Royal Society of Edinburgh, by Dr. Gregory, in March, 1850. Dr. Beatty says:

"The paper is of great value, and should be carefully studied by all who are interested in the success of anæsthetic treatment. He ascribes the injurious effects of the chloroform in ordinary use to the presence of certain volatile oily impurities, which must be removed before it can be safely employed. These oils contain chlorine, have a disagreeable smell, and when inspired or smelt cause distressing headache and sickness. It is, therefore, highly probable that when these symptoms occur, as they do with some individuals, from the use of chloroform of more than average goodness of quality, they depend on the presence of a trace of these poisonous oils. The test which Dr. Gregory recommends for these impurities, is agitation of the chloroform with sulphuric acid, which should be quite colorless, pure, and of the full density of 1.840 at least. This, when agitated with the impure chloroform, becomes yellow or brown, from its action on the oils, which it chars and destroys. Any change of color is easily seen by the contact with the colorless chloroform that floats above. Pure chloroform gives no color to the acid. As this is a subject upon which too much stress cannot be laid, I will beg leave to transcribe Dr. Gregory's instructions for the purification of the adulterated drug:

"The chloroform having been tested as above, and found more or less impure, it is to be agitated with the oil of vitriol (half its volume will be sufficient) and allowed to remain in contact with the acid, of course in a clean, dry, stoppered bottle, and with occasional agitation, till the acid no longer becomes darker in color. As long as the action is incomplete there will be seen after rest at the line of contact a darker ring. When this no longer appears, the chloroform may be drawn off, and for greater security may once more be acted on by a quarter of its volume of the acid, which should now remain colorless. It is now once more to be drawn off, and in a dry, stoppered bottle mixed with a little powdered peroxide of manganese, with which it is gently agitated, and left in contact until the odor of sulphurous acid is entirely destroyed, and the chloroform has acquired a mild, agreeable, fruity smell. It has then only to be drawn off into a proper phial. It will now leave no disagreeable smell when evaporated on the hand.

"Mr. Kemp has observed, in repeating this process for me, the very curious fact, that as soon as the action is complete, but no sooner, the chloroform, tested with the acid in a tube, exhibits a strongly convex surface downwards, where it rests on the pure acid, or what is the same thing, the acid becomes concave at its upper surface. The smallest trace of impurity, not sufficient to effect the density of the chloroform, we have found to render the line of junction horizontal."

